# Different Responses of Soil Environmental Factors, Soil Bacterial Community, and Root Performance to Reductive Soil Disinfestation and Soil Fumigant Chloropicrin

**DOI:** 10.3389/fmicb.2021.796191

**Published:** 2021-12-15

**Authors:** Yu Zhan, Ning Yan, Xinyue Miao, Qiong Li, Changbao Chen

**Affiliations:** Jilin Ginseng Academy, Changchun University of Chinese Medicine, Changchun, China

**Keywords:** reductive soil disinfestation, soil fumigant chloropicrin, bacterial community, environmental factors, ginseng

## Abstract

Reductive soil disinfestation (RSD) and soil fumigant chloropicrin (SFC) are two common agricultural strategies for the elimination of soil-borne pathogens. However, the differences in soil environmental factors, soil bacterial microbiome, and root performance between SFC and RSD are poorly understood. In this study, three soil treatments, untreated control (CK), SFC with 0.5 t⋅ha^–1^ chloropicrin, and RSD with 15 t⋅ha^–1^ animal feces, were compared. We evaluated their effects on soil environmental factors, bacterial community structure, and root activity using chemical analysis and high-throughput sequencing. RSD treatment improved soil composition structure, bacterial diversity, and root performance to a greater extent. Carbon source utilization preference and bacterial community structure were strikingly altered by SFC and RSD practices. Bacterial richness, diversity, and evenness were notably lowered in the SFC- and RSD-treated soil compared with the CK-treated soil. However, RSD-treated soil harbored distinct unique and core microbiomes that were composed of more abundant and diverse potentially disease-suppressive and organic-decomposable agents. Also, soil bacterial diversity and composition were closely related to soil physicochemical properties and enzyme activity, of which pH, available Na (ANa), available Mg (AMg), available Mn (AMn), total Na (TNa), total Ca (TCa), total Cu (TCu), total Sr (TSr), urease (S-UE), acid phosphatase (S-ACP), and sucrase (S-SC) were the main drivers. Moreover, RSD treatment also significantly increased ginseng root activity. Collectively, these results suggest that RSD practices could considerably restore soil nutrient structure and bacterial diversity and improve root performance, which can be applied as a potential agricultural practice for the development of disease-suppressive soil.

## Introduction

Ginseng (*Panax ginseng* C. A. Meyer) is a perennial herb with a long history in the world and is one of the important cash crops (In-Ho and Seung-Ho, 2013). Driven by the rapid development of economy and technology and the shortage of land resources, intensive cropping systems and commercial production mode characterized by continuous monoculture have become an important part of the ginseng industry and have been widely used in the world (Banerjee et al., 2019). However, with the obvious increase of the highly intensive cropping degree and long-term single continuous monoculture trend, ginseng’s dependence on pesticides has been increasing, which poses a serious threat to the environment and product safety. Problems such as degradation of soil quality, serious soil-borne diseases, and inhibition of crop growth are common (Li et al., 2020). This may be caused by decomposable plant residues, root exudates, rainwater leachates, monocropping, and other reasons (Semenov et al., 2020; Vicente et al., 2020). These reasons not only increase the number of pathogens in the soil but also change soil nutrients and plant root activity, which is not conducive to the growth of ginseng and makes them prone to soil-borne diseases (Bulgarelli et al., 2013; Wei et al., 2015). For example, *Cylindrocarpon destructans* was the pathogenic bacteria most seriously causing ginseng root rot, and its incidence could be as high as 70% after continuous implantation (Rahman and Punja, 2005). Worse still, the incidence of ginseng is proportional to the number of years of continuous cultivation, which severely limits the sustainable development of the ginseng industry. Therefore, measures to overcome or alleviate soil degradation and soil-borne diseases have become an urgent concern.

Soil fumigation is the most widely used method to control soil-borne pathogens (Strauss and Kluepfel, 2015). Chloropicrin is one of the commonly used soil fumigants (Jackson, 1934). Studies have shown that the chloropicrin fumigant can kill more than 85% of bacteria, fungi, and actinomycetes in soil (Gullino et al., 2002) and also has certain control effects on weeds and nematodes (Haar et al., 2003; Yan et al., 2012). However, with the increasing concern for the sustainable development of agriculture and human health, the traditional chemical fumigants have been gradually phased out. Therefore, screening a non-chemical, green, efficient and practical method that can effectively replace soil fumigation is the focus of research. In this case, reductive soil disinfestation (RSD), also known as biological soil disinfestation (BSD) or anaerobic soil disinfestation (ASD), independently developed by scientists from Japan (Momma et al., 2013) and Netherlands (Blok et al., 2000), has attracted widespread attention. It is a method that uses ecological principles to treat soil before plants are planted, by enriching soil organic carbon sources to create soil environments that are favorable for crop growth but unfavorable for pathogen growth, making the beneficial microorganisms in the soil occupy a favorable ecological niche and thus inhibiting the growth of harmful microbes, to reduce the incidence rate of soil disease spread and to increase crop yield (Di Gioia et al., 2017; Ueki et al., 2018; Zhou et al., 2019). At present, it has been applied to a variety of crop production systems, such as *Salvia miltiorrhiza* Bunge (Yang et al., 2021), *Lilium brownii* var. *viridulum* (Zhou et al., 2019), and *Panax notoginseng* (Burk.) F. H. Chen (Li et al., 2019), and has been confirmed as an effective practice for extensive control of plant pathogens.

Soil microorganisms are the most active component in the soil microecological environment. They maintain multiple ecological processes such as decomposition of soil organic matter, formation of humus, material circulation, and energy exchange between plants and the environment and play an indicator role in soil fertility and soil environment (Goldford et al., 2018). At the same time, most of the soil enzymes are secreted by soil microorganisms (Ahmed et al., 2018), while a few are secreted by plant roots and soil animals (Burns, 1982). These enzymes are closely related to soil nutrients (Allison et al., 2010), which together constitute the soil microecological environment and affect the root activity of plants (Bowles et al., 2014). Recently, the results showed that both chemical soil disinfestation (CSD) and RSD have excellent ability to inhibit pathogens and strikingly alter the bacterial preference for carbon source utilization preference and bacterial community structure (Zhao et al., 2018; Huang et al., 2019). Soil fumigant chloropicrin (SFC) is the most widely used technique in CSD. However, knowledge regarding the influence of SFC and RSD on soil bacterial microbiota is still limited. In addition, soil microbial community is sensitive to changes in the external environment (Balser and Firestone, 2005), and changes in the soil oxygen environment will inevitably affect the diversity, composition, and structure of soil bacterial community. Studies have shown that soil aeration can effectively improve the number of soil microorganisms and soil enzyme activities (Li et al., 2016). At the same time, the roots of plants also need oxygen for respiration (Armstrong et al., 2019). However, RSD treatment mainly creates anaerobic conditions by adding organic matter and then flooding the soil with water and covering it with a plastic film (Nevein et al., 2007). Therefore, it is particularly important to identify the changes in soil bacterial community, soil enzyme activity, and soil nutrients in SFC and RSD in an anaerobic environment.

Due to the differences in materials used and mechanisms involved between SFC and RSD, we hypothesized that (1) SFC and RSD differentially impact the soil bacterial community structure and diversity; (2) RSD could increase the number of antagonistic bacteria and root activity, compared with CSD; and (3) the anaerobic environment may indirectly affect plant root activity by changing the soil microecological environment. To test these hypotheses, chemical analysis and high-throughput sequencing technology were used to study the relationship between the ginseng rhizosphere bacterial community and soil environmental factors under different anaerobic conditions, and ginseng root activity was measured. The relationship between the change in soil bacterial community and ginseng root activity was further analyzed.

## Materials and Methods

### Field Experiment Description and Design

The field experiment was performed in Zuojia Town, Changyi District, Jilin City, Jilin Province, China (44°02′N, 126°15′E, 237-m altitude), which is characterized by a temperate continental monsoon climate with a mean annual temperature and precipitation of 5.8°C and 550 mm, respectively. There are an average of 2,530 h of sunshine and approximately 128 growth days per year. The soil is dark-brown forest soil. Before the experiment, ginseng was planted continuously for 3 years, and the soil sickness was serious. Physical and chemical properties of soils were as follows: pH value 5.81; soil electric conductivity (EC) 70.45 μs⋅cm^–1^; available Na (ANa) 0.1279 mg⋅g^–1^; available Mg (AMg) 0.4074 mg⋅g^–1^; available K (AK) 0.2043 mg⋅g^–1^; available Ca (ACa) 0.9485 mg⋅g^–1^; available Mn (AMn) 0.06725 mg⋅g^–1^; available Fe (AFe) 0.4363 mg⋅g^–1^; available Cu (ACu) 0.00322 mg⋅g^–1^; available Sr (ASr) 0.01871 mg⋅g^–1^; total Na (TNa) 3.4875 mg⋅g^–1^; total Mg (TMg) 0.6475 mg⋅g^–1^; total K (TK) 20.15 mg⋅g^–1^; total Ca (TCa) 0.47725 mg⋅g^–1^; total Mn (TMn) 0.55625 mg⋅g^–1^; total Fe (TFe) 13.64 mg⋅g^–1^; total Cu (TCu) 0.01775 mg⋅g^–1^; total Zn (TZn) 0.0665 mg⋅g^–1^; and total Sr (TSr) 0.01175 mg⋅g^–1^.

Three treatments were employed in this study: (1) untreated control (CK), soil was untreated; (2) SFC, soil was injected with 0.5 t⋅ha^–1^ chloropicrin (Dalian Lufeng Chemical Co., Ltd., Liaoning, China) and covered with a 0.04-mm blue plastic film; and (3) RSD, soil was added with 15 t⋅ha^–1^ animal feces (chicken feces, cow feces, and pig feces = 1:1:1), irrigated to 100% water holding capacity, and covered with a 0.04-mm blue plastic film. Each treatment contained three replicates, and each replicate covered an area of 30 m^2^ (2 m × 15 m) in a randomized complete block design. The treatments, except for the control, lasted for a period of 4 weeks under strict anaerobic conditions. The soil temperature ranged from 30 to 40°C. After the 4-week treatment, the plastic films were removed, and the soil was overturned after 2–3 days of natural drying. Ginseng transplanting was carried out on October 20, 2019, and 2-year-old healthy ginseng seedlings of similar size were transplanted. During the experiment, the field management measures were consistent with local production practice.

### Sample Collection and Processing

Soil samples and plants were collected during the first harvest period (October 1, 2020). Twenty soil sampling points were set up according to the “S” shape in each treatment, and five soil sampling points were mixed into one composite sample after the soil was collected. This method meant that four composite soil samples were obtained from each treatment. As a result, a total of 12 soil composite samples were collected from 3 treatments at a depth of 0–20 cm. Subsequently, roots and impurities were removed from soils. One fraction was air-dried and ground to pass through a 2-mm mesh size sieve for subsequent soil property and enzyme activity analyses, while the remaining fraction was taken to the laboratory in cool boxes with ice bags and stored at low temperature (−80°C) for high-throughput sequencing analysis. At the same time, six plants were randomly selected from each treatment and labeled. The aboveground and underground parts of the plants were separated and washed gently, and the roots were dried with absorbent paper and used for an analysis of root activity.

### Soil Property Analyses

Soil pH was determined by a pH meter (soil-to-water ratio was 1:5). Soil EC was measured by a conductivity meter (soil-to-water ratio was 1:5). The metal ions ANa, AMg, AK, ACa, AMn, AFe, ACu, available Zn (AZn), and ASr were extracted by the M_3_ method and determined by inductively coupled plasma–optical emission spectrometry (ICP-OES). The metal ions TNa, TMg, TK, TCa, TMn, TFe, TCu, TZn, and TSr were determined by ICP-OES with a concentrated nitric acid method.

### Soil Enzyme Activity Analysis

Important enzymes involved in soil nutrient cycle processes and microbial metabolism include urease (S-UE), acid phosphatase (S-ACP), sucrase (S-SC), catalase (S-CAT), and laccase (SL). These enzymes were measured using a kit produced by the company Solarbio. Enzyme activity was determined using 96-well microtiter plates and followed the product manual provided by Solarbio. Each sample was repeated five times. S-UE activity was defined as 1 μg of NH_3_-N produced per gram of soil per day. S-ACP activity was defined as 1 nmol of phenol released per gram of soil per day at 37°C as one enzyme activity. S-SC activity was defined as 1 mg of reducing sugar per gram of soil per day at 37°C. S-CAT activity was defined as catalytic degradation of 1 μmol of H_2_O_2_ per gram of air-dried soil sample per day. SL activity was defined as the amount of enzyme required to generate 1 nmol of 2,2′-azino-bis(3-ethylbenzothiazoline-6-sulfonic acid (ABTS) free radical per minute per gram of soil.

### Soil DNA Extraction and Sequencing

Total DNA was extracted from 0.5 g of soil per replicate, using the E.Z.N.A.^®^ Soil DNA Kit (Omega Bio-tek, Norcross, GA, United States) according to the manufacturer’s instructions. The integrity of DNA was validated by 1% agarose gel electrophoresis. The DNA concentration and purity were determined with a NanoDrop 2000 UV–vis spectrophotometer (Thermo Fisher Scientific, Wilmington, DE, United States). An Illumina MiSeq PE300 platform (Illumina, United States) was used to measure diversity and composition of bacterial community. The universal 16S rRNA gene primers 338F (5′-ACTCCTACGGGAGGCAGCA-3′) and 806R (5′-GGACTACHVGGGTWTCTAAT-3′) were chosen for the amplification and subsequent high-throughput sequencing of the polymerase chain reaction (PCR) products. Each 20-μl PCR mixture contained 4 μl of FastPfu buffer (5 × Transgen), 2 μl of dNTPs (2.5 mM), 0.8 μl of forward primer (5 μM), 0.8 μl of reverse primer (5 μM), 0.4 μl of TransStart FastPfu DNA polymerase, 10 ng of template DNA, and 0.2 μl of ddH_2_O. The PCR amplification of 16S rRNA gene was performed as follows: initial denaturation at 95°C for 3 min, followed by 27 cycles of denaturing at 95°C for 30 s, annealing at 55°C for 30 s and extension at 72°C for 45 s, a single extension at 72°C for 10 min, and ending at 4°C. The raw reads were deposited into the National Center for Biotechnology Information (NCBI) Sequence Read Archive (SRA) database (accession number: PRJNA765568).

### Root Activity

The obtained roots were carefully washed, and the aboveground parts were removed from the stem base. The fresh plant root sample was used to assess root activity by the 2,3,5-triphenyl tetrazolium chloride (TTC) method. The oxidation state of TTC is colorless in itself. We soaked the roots in a TTC aqueous solution, and TTC entered the root cells. This test is based on dehydrogenase in live roots (especially succinate dehydrogenase in mitochondria) reducing colorless TTC to red triphenyl formazan. The latter compound is then extracted after a fixed incubation period and by spectrophotometry at 485 nm. Root activity is expressed as the mass triphenyl formazan produced in milligrams per gram fresh root per hour.

### Statistical Analysis

We used FLASH V1.2.11 software filtering under the condition of certain filtering of the original data and quality control in accordance with QIIME V1.9.1 high-quality data. Sequence length <200 bp, average quality <20 bp or containing a small number of bases, effectively using the RDP Classifier algorithm and SILVA database to chimerically detect and remove, after similarity, >97% of the sequences classified into the same operating classification unit (OTU) for each filtered OTU on behalf of the sequence and annotation. OTUs were used to generate rarefaction curves and Shannon–Wiener curves. Bacterial Shannon diversity, richness (Sobs), and evenness (shannoneven) and coverage were calculated based on the rarefied OTU table at a depth of 60,000 sequences per sample. Principal coordinates analysis (PCoA) and hierarchical cluster analysis were conducted using the Bray–Curtis distance matrix. Permutational multivariate analysis of variance (PERMANOVA) (adonis) and permutational analysis of multivariate dispersions (PERMDISP) were applied to investigate the bacterial community differences among treatments and the homogeneity of replicate dispersions, respectively. The treatment-shared OTUs and treatment-unique OTUs were analyzed by retaining the OTUs that consistently appeared in the four biological replicates for each treatment. The shifts in the relative abundance of the bacterial families were displayed by a heat map. The correlation between soil environmental factors (soil properties and soil enzyme activity) and bacterial community structure was analyzed by redundancy analysis (RDA). The Pearson correlation coefficient of the top 20 abundant bacterial families and soil environmental factors is displayed on the heat map.

Experimental data were organized using Microsoft Excel. The statistical software SPSS 21.0 was used for one-way analysis of variance (ANOVA), and the *p*-value threshold of <0.05 was used to characterize significant differences between the three groups of data. All graphics are built using the drawing software GraphPad Prism 8.01 and the Majorbio platform.

## Results

### Physicochemical Properties of Rhizosphere Soils

One-way ANOVA revealed that most of the soil properties were changed significantly under different treatments except AMg (Table 1). Soil pH value, EC, AK, AFe, AZn, TNa, TFe, TCu, and TZn were increased but ACa, ASr, and TMn was decreased by both RSD and SFC treatments, as compared with CK. In particular, other indexes significant differences except soil pH value, AFe, and AZn, and AK, ACa, ASr, TNa, TFe, TCu, and TZn content showed SFC > RSD, and EC and TMn showed RSD > SFC. Furthermore, RSD and SFC treatments were slightly different compared with CK treatment. Soil pH value, EC, ANa, AK, AMn, AZn, TNa, TFe, TCu, and TZn were increased but ACa, ASr, TMg, TK, TCa, TMn, and TSr were decreased by the SFC treatment. Soil pH value, EC, AK, ACu, AZn, TNa, TMg, TK, TCa, TFe, TCu, TZn, and TSr were increased but ACa, ASr, and TMn were decreased by the RSD treatment.

**TABLE 1:** Physicochemical properties and nutrient content of the soil samples under different treatments.

		Soil physicochemical properties and nutrient content			
	**Parameter 1**	**pH value**	**EC (μs⋅cm^–1^)**	**AK (mg⋅g^–1^)**	**TK (mg⋅g^–1^)**
Treatment	CK	5.180 b	47.035 c	0.151 c	20.800 b
	SFC	6.110 a	131.775 b	0.179 a	16.193 c
	RSD	6.123 a	159.025 a	0.167 b	21.685 a
	**Parameter 2**	**ANa (mg⋅g^–1^)**	**AMg (mg⋅g^–1^)**	**ACa (mg⋅g^–1^)**	**AMn (mg⋅g^–1^)**
Treatment	CK	0.129 b	0.477 a	1.037 a	0.061 b
	SFC	0.136 a	0.482 a	0.969 b	0.084 a
	RSD	0.128 b	0.487 a	0.942 c	0.061 b
	**Parameter 3**	**AFe (mg⋅g^–1^)**	**ACu (mg⋅g^–1^)**	**AZn (mg⋅g^–1^)**	**ASr (mg⋅g^–1^)**
Treatment	CK	0.353 b	0.004 b	0.008 b	0.021 a
	SFC	0.407 a	0.004 b	0.010 a	0.019 b
	RSD	0.402 a	0.005 a	0.010 a	0.018 c
	**Parameter 4**	**TNa (mg⋅g^–1^)**	**TMg (mg⋅g^–1^)**	**TCa (mg⋅g^–1^)**	**TMn (mg⋅g^–1^)**
Treatment	CK	3.615 c	1.031 b	0.664 b	0.595 a
	SFC	4.315 a	0.356 c	0.412 c	0.477 c
	RSD	4.145 b	1.103 a	0.715 a	0.544 b
	**Parameter 5**	**TFe (mg⋅g^–1^)**	**TCu (mg⋅g^–1^)**	**TZn (mg⋅g^–1^)**	**TSr (mg⋅g^–1^)**
Treatment	CK	14.500 c	0.019 c	0.062 c	0.019 b
	SFC	22.155 a	0.020 b	0.092 a	0.009 c
	RSD	15.015 b	0.023 a	0.081 b	0.019 a

*Data in the table represent three repeated means. There were significant differences between different letters in different treatments (p < 0.05).*

### Enzyme Activities of Rhizosphere Soils

One-way ANOVA revealed that the activities of S-ACP, S-CAT, and SL were significantly lower than those in the CK treatment, except S-UE and S-SC, and they were in the order of CK > RSD > SFC (Table 2). In addition, compared with those in the RSD, the activities of five soil enzymes changed significantly in the SFC treatment. In particular, activities of five soil enzymes in the SFC treatment was significantly lower than those in the RSD treatment.

**TABLE 2:** Enzyme activities of the soil samples under different treatments.

Parameter	S-UE (U⋅g^–1^)	S-ACP (nmol⋅d⋅g^–1^)	S-SC (U⋅g^–1^)	S-CAT (μmol⋅d⋅g^–1^)	SL (nmol⋅min⋅g^–1^)
CK	3,692.63 b	31,244.57 a	24.46 a	24.79 a	137.95 a
SFC	3,809.80 b	18,222.54 c	12.58 b	17.77 c	93.92 c
RSD	4,739.95 a	26,276.62 b	21.50 a	22.59 b	100.29 b

*Data in the table represent three repeated means. There were significant differences between different letters in different treatments (p < 0.05).*

### Diversity, Composition, and Structure of Soil Bacterial Community

#### Bacterial α-Diversity and β-Diversity

**FIGURE 1:**
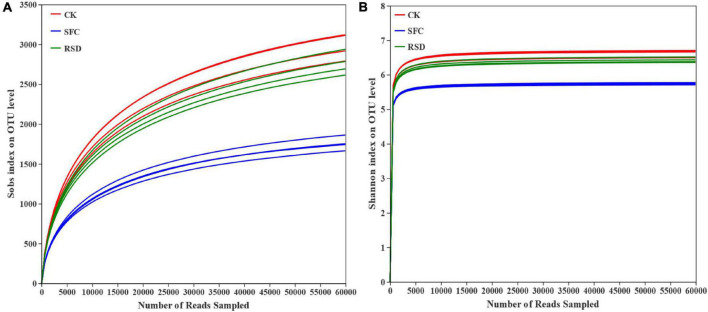
Rarefaction curves **(A)** and Shannon–Wiener curve **(B)** of the bacterial communities under different treatments.

A total of 870,064 high-quality 16S rRNA gene sequences were obtained from 12 soil samples in 3 different treatments (ranging from 70,712 to 74,932 across different samples) in this study, which were obtained from MiSeq sequencing. These sequences were distributed among 5,401 different OTUs at 97% similarity. The rarefaction curve showed that the sequencing work was relatively comprehensive in covering the bacterial diversity, as the rarefaction curves tended to approach saturation (Figure 1A). The Shannon curve indicated that the data set from the diversity analysis was large enough to reflect the bacterial diversity information of samples (Figure 1B).

**FIGURE 2:**
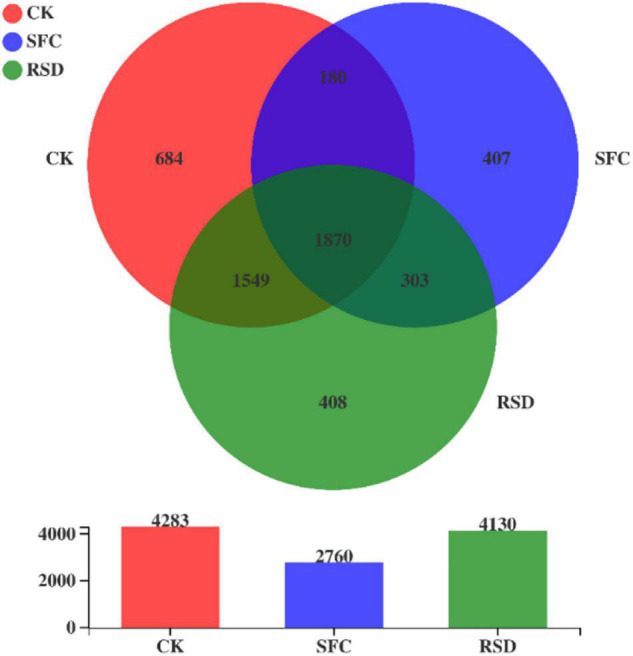
Venn diagram of the bacterial communities under different treatments.

To some extent, the RSD treatment significantly (*p* < 0.05) decreased the observed bacterial richness (Sobs), Shannon diversity, and evenness (shannoneven) compared with the CK treatment (Table 3). In contrast, the RSD treatment significantly (*p* < 0.05) increased the bacterial richness, diversity, and evenness and decreased bacterial coverage compared with the SFC treatment. The numbers of bacterial OTUs accumulated to 1,870, among the 3 treatments (Figure 2).

**TABLE 3:** Soil bacterial richness, diversity, evenness, and coverage under different treatments.

Treatment	Sobs	Shannon	Shannoneven	Coverage
CK	2,986.8 ± 160.58 a	6.6383 ± 0.10 a	0.82967 ± 0.01 a	0.99032 ± 0.00 b
SFC	1,758.0 ± 74.33 c	5.7475 ± 0.03 c	0.76928 ± 0.00 c	0.99478 ± 0.00 a
RSD	2,751.8 ± 136.39 b	6.4211 ± 0.07 b	0.81082 ± 0.00 b	0.99053 ± 0.00 b

*Values (means ± SD, n = 4) within the same column followed by different letters are significantly different at p < 0.05 according to the Fisher least-significant difference (LSD) post hoc test.*

#### Bacterial Community Composition

**FIGURE 3:**
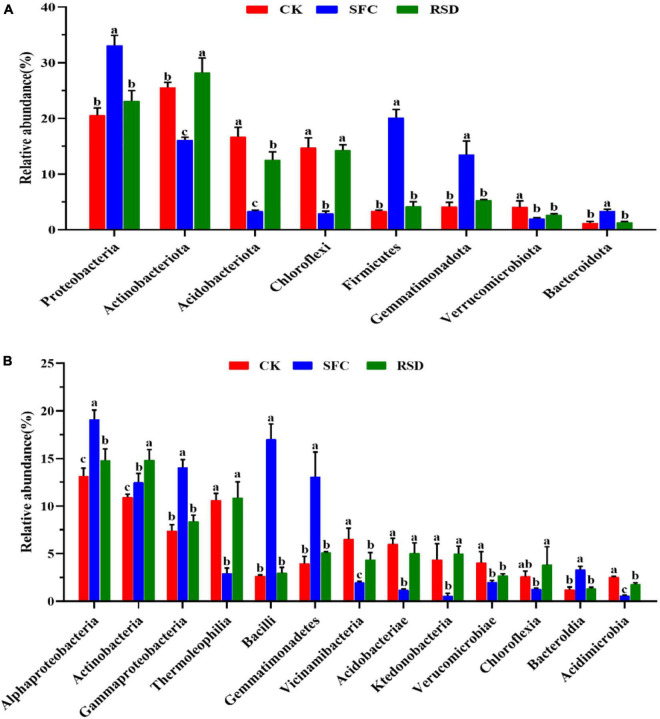
Relative abundances of dominant bacterial phylum **(A)** and classes **(B)** in different treatments. Error bars indicate the standard errors of the means of four replicates. The letters indicate significant difference at *p* < 0.05 according to one-way analysis of variance (ANOVA) among treatments.

**FIGURE 4:**
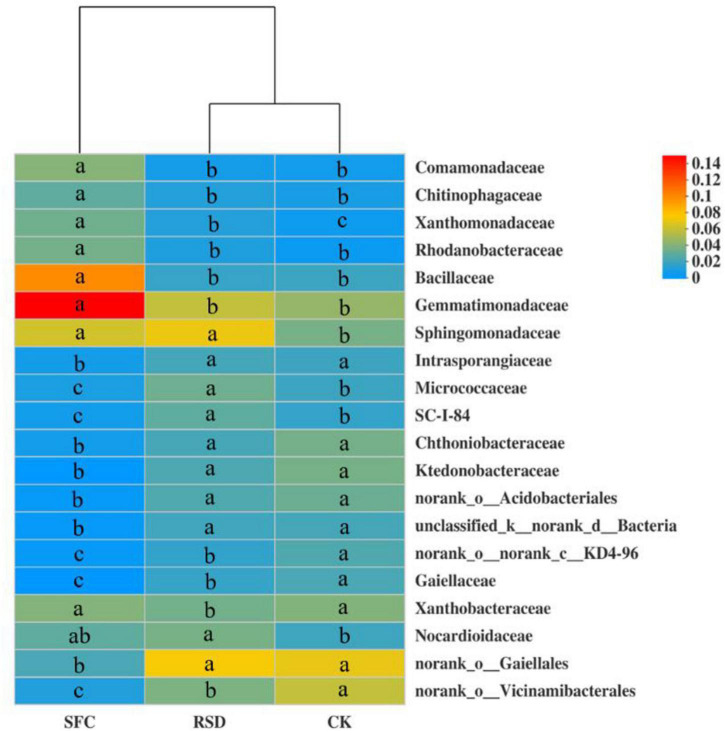
Heat map displaying the average relative abundance of the top 20 bacterial families for all treatments. The key from blue to red represents the least abundant to most abundant in each row for a given family. The letters above the treatments indicate significant difference at *p* < 0.05 among treatments according to one-way analysis of variance (ANOVA).

Soil fumigant chloropicrin and RSD treatments significantly altered the soil bacterial community composition from the family to phylum levels, in particular the top 20 bacterial families (Figures 3, 4). Compared to those in the CK soil, the relative abundances of *Comamonadaceae*, *Chitinophagaceae*, *Xanthobacteraceae*, *Rhodanobacteraceae*, *Bacillaceae*, *Gemmatimonadaceae*, *Sphingomonadaceae*, and *Nocardioidaceae* increased significantly in the SFC-treated soils, whereas the relative abundances of *Intrasporangiaceae*, *Micrococcaceae*, *Chthoniobacteraceae*, *Ktedonobacteracea*, and *Gaiellales* decreased significantly (*p* < 0.05). However, the relative abundances of *Sphingomonadaceae*, *Micrococcaceae*, and *Nocardioidaceae* increased markedly in the RSD-treated soil, whereas the relative abundances of *Gaiellales* and *Xanthobacteraceae* decreased significantly (*p* < 0.05) (Figure 4).

**FIGURE 5:**
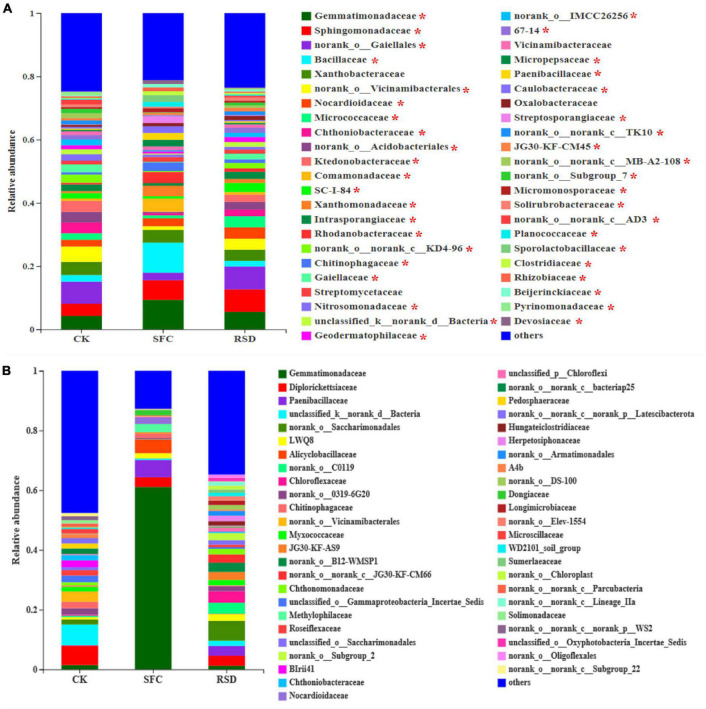
Relative abundance of bacterial families (RA > 1%) in the shared OTUs **(A)** and unique OTUs **(B)**. The * above the taxon in the shared OUT indicates significant difference at *p* < 0.05 among treatments according to one-way analysis of variance (ANOVA).

Moreover, SFC and RSD treatments also considerably shaped the shared and unique bacterial microbiomes (Table 4 and Figure 5). The number of shared OTUs was 1,870, which accounted for 34.62% of the total retained OTUs (5,401) and 43.66, 67.75, and 45.28% of the retained OTUs for CK-, SFC-, and RSD-treated soils, respectively (Table 4). The majority of the shared OTUs was classified into 46 bacterial families, and the relative abundance of 42 families shifted considerably (*p* < 0.05) among treatments (Figure 5A). In particular, the families *Gemmatimonadaceae*, *Bacillaceae*, *Comamonadaceae*, *Xanthomonadaceae*, *Rhodanobacteraceae*, *Chitinophagaceae*, *Micropepsaceae*, *Paenibacillaceae*, *Caulobacteraceae*, *Streptosporangiaceae*, *Micromonosporaceae*, *Planococcaceae*, *Sporolactobacillaceae*, *Clostridiaceae*, *Rhizobiaceae*, and *Beijerinckiaceae* were significantly enriched in the SFC treatment, whereas the families *Micrococcaceae*, *Chthoniobacteraceae*, *Ktedonobacteraceae*, *Intrasporangiaceae*, *Gaiellaceae*, *Nitrosomonadaceae*, *Geodermatophilaceae*, *Solibacteraceae*, and *Solirubrobacteraceae* were significantly (*p* < 0.05) depleted. The families *Sphingomonadaceae*, *Nocardioidaceae*, *Micrococcaceae*, *Intrasporangiaceae*, *Geodermatophilaceae*, *Oxalobacteraceae*, and *Solirubrobacteraceae* were significantly enriched in the SFC treatment, whereas the families *Bacillaceae* and *Xanthobacteraceae* were significantly (*p* < 0.05) depleted.

**TABLE 4:** The number of unique OTUs for each treatment and overlapped OTUs for every pair of treatments per 60,000 sequences.

Treatment	CK	SFC	RSD
CK	***684***		
SFC	*180*	***407***	
RSD	*1,549*	*303*	***408***
Shared OTUs	1,870	1,870	1,870
Total OTUs	4,283	2,760	4,130

*Values in bold italics represent unique OTUs in each treatment, and italics represent overlapped OTUs between two treatments. Only the OTUs present in four biological replicates of each treatment were retained for analyses.*

The number of total treatment-unique OTUs was 1,499 and occupied 27.75% of the total retained OTUs. The number of OTUs unique to the CK, SFC, and RSD treatments accounted for 15.97, 14.75, and 9.88% of the retained OTUs, respectively (Table 4). Furthermore, the sequences that belonged to the treatment-unique OTUs were affiliated into 48 bacterial families, and the CK-, SFC-, and RSD-treated soils harbored 39, 24, and 42 families, respectively (Figure 5B and Table 5), suggesting that the RSD treatment is capable of reassembling a more diverse unique microbiome than the SFC treatment. In particular, the families *Methylophilaceae*, *Nocardioidaceae*, and *Dongiaceae* were only observed in the SFC-treated soil, and the *norank_o__B12-WMSP1*, *norank_o__Subgroup_2*, *Herpetosiphonaceae*, and *norank_o__Armatimonadales* were found only in the RSD-treated soil. Furthermore, the families *Alicyclobacillaceae* and *norank_o__Oligoflexales* were observed only in the SFC- and RSD-treated soils, whereas *BIrii41*, *norank_o__norank_c__norank_p__WS2*, and *norank_o__norank_c__Subgroup_22* were found in the SFC- and RSD-treated soils. Despite the unique bacterial families that appeared in the RSD treatment, several different families were enriched after each of the soil disinfestation treatment. For instance, the families *Gemmatimonadaceae*, *Paenibacillaceae*, *Alicyclobacillaceae*, *Methylophilaceae*, *Nocardioidaceae*, and *Dongiaceae* were enriched in the SFC-treated soil, while the families *Chloroflexaceae*, *Hungateiclostridiaceae*, *Herpetosiphonaceae*, *Longimicrobiaceae*, and *Sumerlaeaceae* increased largely in the RSD-treated soil.

**TABLE 5:** Relative abundance of bacterial families in the unique OTUs in each treatment.

Bacterial families	Treatment		
	CK	SFC	RSD
Gemmatimonadaceae	1.46	61.07	1.19
Diplorickettsiaceae	6.50	3.32	3.38
Paenibacillaceae	0.13	5.77	3.32
unclassified_k__norank_d__Bacteria	6.96	0.46	1.72
norank_o__Saccharimonadales	1.66	0.07	6.63
LWQ8	0.80	1.72	2.25
Alicyclobacillaceae	ND	4.64	0.13
norank_o__C0119	0.46	0.07	3.71
Chloroflexaceae	0.33	ND	3.85
norank_o__0319-6G20	2.19	0.33	1.66
Chitinophagaceae	2.19	1.46	0.27
norank_o__Vicinamibacterales	3.45	ND	0.07
Myxococcaceae	1.59	0.07	1.79
JG30-KF-AS9	0.20	0.46	2.65
norank_o__B12-WMSP1	ND	ND	3.12
norank_o__norank_c__JG30-KF-CM66	0.20	ND	2.79
Chthonomonadaceae	1.06	ND	1.86
unclassified_o__Gammaproteobacteria_Incertae_Sedis	2.25	ND	0.53
Methylophilaceae	ND	2.72	ND
Roseiflexaceae	1.79	ND	0.86
unclassified_o__Saccharimonadales	0.99	ND	1.53
norank_o__Subgroup_2	ND	ND	2.39
BIrii41	2.32	ND	ND
Chthoniobacteraceae	1.79	ND	0.40
Nocardioidaceae	ND	2.19	ND
unclassified_p__Chloroflexi	0.33	0.40	1.39
norank_o__norank_c__bacteriap25	1.86	ND	0.27
Pedosphaeraceae	1.66	0.20	0.20
norank_o__norank_c__norank_p__Latescibacterota	1.72	ND	0.20
Hungateiclostridiaceae	0.13	0.20	1.53
Herpetosiphonaceae	ND	ND	1.79
norank_o__Armatimonadales	ND	ND	1.66
A4b	1.26	ND	0.40
norank_o__DS-100	0.13	ND	1.53
Dongiaceae	ND	1.66	ND
[-0.8pt] Longimicrobiaceae	0.13	ND	1.46
norank_o__Elev-1554	0.07	ND	1.46
Microscillaceae	1.39	0.07	0.07
WD2101_soil_group	0.33	0.07	1.13
Sumerlaeaceae	0.27	0.07	1.13
norank_o__Chloroplast	0.07	0.13	1.19
norank_o__norank_c__Parcubacteria	1.19	0.07	0.07
norank_o__norank_c__Lineage_IIa	0.07	ND	1.26
Solimonadaceae	1.06	ND	0.20
norank_o__norank_c__norank_p__WS2	1.26	ND	ND
unclassified_o__Oxyphotobacteria_Incertae_Sedis	0.07	ND	1.19
norank_o__Oligoflexales	ND	0.07	1.06
norank_o__norank_c__Subgroup_22	1.06	ND	ND
others	47.61	12.73	34.75

*ND indicates not detected.*

#### Bacterial Community Structure

**FIGURE 6:**
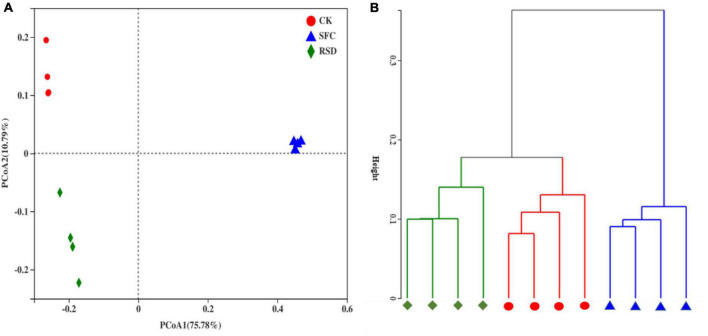
Principal coordinates analysis (PCoA) **(A)** and hierarchical cluster analysis **(B)** of the bacterial community based on the Bray–Curtis distance in the soil samples under different treatments.

In the PCoA, the principal coordinates explained 75.78 and 10.79% of the total variation in bacterial communities (Figure 6A). Bacterial communities in the SFC, RSD, and CK treatments distinctly clustered into three groups, indicating that anaerobic treatment and fumigant treatment restructured the bacterial community in soil. However, the soil bacterial community structures of the three treatments were well grouped and separated from each other; the bacterial community structures of CK and RSD treatments were similar, indicating that the SFC treatment significantly (PERMANOVA, *p* < 0.001) altered the soil bacterial community structure. Similarly, hierarchical cluster analysis further showed that the CK treatment had an effect on the soil bacterial community structure similar to that of the RSD treatment, which together formed a cluster distinct from that of the SFC treatment (Figure 6B).

### Correlation Between Soil Physicochemical Properties or Enzyme Activities and Bacterial Community Composition at the Family Level

**FIGURE 7:**
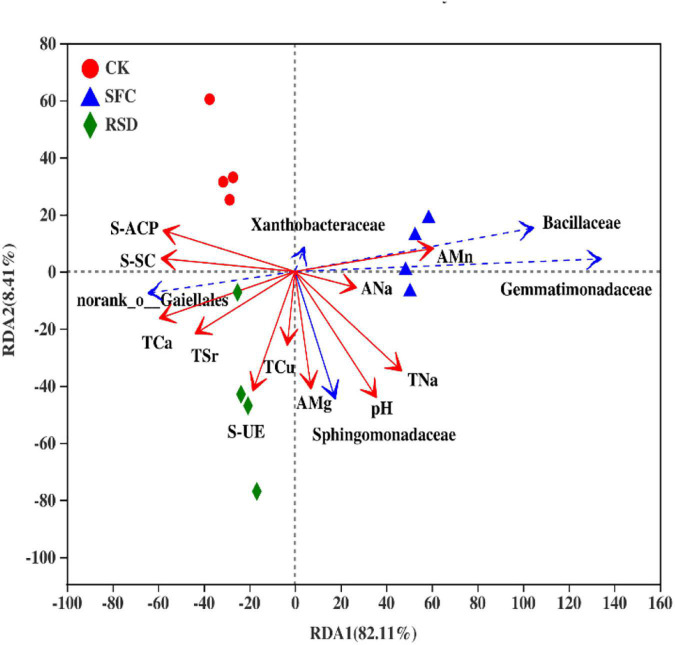
Redundancy analysis (RDA) of bacterial communities based on relative abundances at the family level and soil properties in individual samples.

Environmental factors were selected by the functions of envfit (permu = 999) and vif.cca, and the environmental factors with *p* > 0.05 or variance inflation factor (VIF) >10 were removed from the following analysis. The VIF values of EC, AK, ACa, AFe, ACu, AZn, ASr, TMg, TK, TMn, TFe, TZn, S-CAT, and SL were higher than 10 and removed (Table 6). The correlation between soil environmental factors (soil properties and soil enzyme activity) and bacterial community structure was analyzed by RDA (Figure 7). For soil bacterial communities, soil S-ACP, S-SC, TCa, TSr, S-UE, and TCu were positively correlated with the relative abundances of *norank o_ Gaiellales*; soil TSr, S-UE, TCu, AMg, pH, TNa, ANa, and AMn were positively correlated with the relative abundances of *Sphingomonadaceae*; soil pH, TNa, ANa, and AMn were positively correlated with the relative abundances of *Gemmatimonadaceae* and *Bacillaceae*; soil S-ACP, S-SC, TNa, ANa, and AMn were positively correlated with the relative abundances of *Xanthobacteraceae*. However, soil S-ACP and S-SC were negatively correlated with the relative abundances of *Sphingomonadaceae*, *Gemmatimonadaceae*, and *Bacillaceae*; soil AMn, ANa, TNa, and pH were negatively correlated with the relative abundances of *norank o_ Gaiellales*; soil TCa, TSr, S-UE, TCu, AMg, and pH were negatively correlated with the relative abundances of *Xanthobacteraceae*.

**TABLE 6:** Environmental factor VIF value.

Item	Treatment										
	pH	ANa	AMg	AMn	TNa	TCa	TCu	TSr	S-UE	S-ACP	S-SC
VIF	5.74	1.25	4.38	2.16	1.56	2.76	1.09	2.36	1.06	7.14	7.26

### Ginseng Root Morphology and Activity

Plant root is an active absorbing organ and synthetic organ; its growth and activity directly affect the level of plant nutrition and yield. Compared with CK, SFC, and RSD treatments promoted root morphology and activity (Figures 9, 10). However, in root morphology, SFC treatment was significantly better than RSD treatment, which showed that the ginseng fibrous root was dense; tuber expansion was normal, and disease spots were reduced (Figure 9). In root activity, triphenyl formazan content of RSD treatment was significantly higher than that of SFC treatment, and triphenyl formazan content was 2.38 times that of SFC treatment (Figure 10). In general, the root morphology of ginseng treated with SFC was better, and the root activity of ginseng treated with RSD was stronger.

## Discussion

### Reductive Soil Disinfestation and Soil Fumigant Chloropicrin Altered the Bacterial Community Structure and Diversity Differently

Numerous studies have reported that soil microbiomes are considerably influenced by RSD and SFC (Huang et al., 2016a; Zhang et al., 2021; Zhu et al., 2021). In this study, we observed that SFC treatment significantly modified the soil bacterial community structure compared with the CK treatment, which is consistent with several other studies that showed remarkable shifts in soil bacterial communities after chemical fumigation (Yan et al., 2019; Sun et al., 2020). This is likely due to the direct toxicity of chloro-nitromethane and nitromethane generated by chloropicrin degradation, which has a broad-spectrum biocidal activity against indigenous soil microbes (Wilhelm, 1997; Gan et al., 2000). The destructive influences on original soil microbial community during chloropicrin fumigation may prevent the successful recovery of soil bacterial communities (Fang et al., 2018).

Moreover, the impact of RSD on microbial activity, community structure, and functional groups such as pathogenic and beneficial microorganisms has been extensively investigated in the past two decades (Sun et al., 2016; Ueki et al., 2018). Previous studies showed distinct differences in bacterial community structure after incorporation of different organic substrates (Sun et al., 2016; Zhou et al., 2019). For this study, bacterial communities treated with SFC and RSD were well grouped and separated from each other and considerably shaped the shared and unique bacterial microbiomes. It is possibly attributed to the differences in the degradability and carbon composition among the various organic substrates (Liu et al., 2016; Tan et al., 2019).

The degradability of materials is an inherent property that may induce profound changes in the composition and structure of the microbial community (Liu et al., 2016). In SFC and RSD treatments, soil bacterial α-diversity and β-diversity changed notably. It is assumed that this phenomenon was mainly due to the degradability of the materials (pure chemicals of chloropicrin vs. organic residues) used rather than the disinfestation methods used. It is likely that pure chemicals of chloropicrin are more easy to decompose, thereby killing all the microorganisms, and the soil microbial community may not be able to recover or may need a long time to recover, leading to a considerable decline in bacterial richness, diversity, and evenness (Zhao et al., 2018). The results are consistent with those of Zhang et al. (2021) that is, soil treated with fresh chicken manure not only improved the respiration rate of soil microorganisms but also shortened the recovery time of beneficial soil microorganisms and increased taxonomic diversity. This is possibly because fresh chicken manure is rich in organic matter and will form dissimilar soil bacterial microbiomes by stimulating different bacterial taxa to participate in the decomposition and reductive processes under anaerobic conditions.

### Relationships Between Soil Environmental Factors and Bacterial Community

Soil environmental factors play a critical role in bacterial community; in particular, soil physicochemical properties and enzyme activities have been found to greatly affect its bacterial community (Lin et al., 2021; Zhao et al., 2021). However, soil salinization and acidification are known as two characteristics of soil degradation in intensive agricultural systems (Shi et al., 2009); a degraded soil environment always facilitates the proliferation of soil-borne pathogens (Liu et al., 2014). In the present study, pH exhibited a significant increase in SFC- and RSD-treated soil, especially in RSD-treated soil, compared to that of CK-treated soil, which is consistent with previous reports (Meng et al., 2019). Likewise, further analysis showed a positive correlation between soil pH and the relative abundances of *Sphingomonadaceae*, *Gemmatimonadaceae*, and *Bacillaceae* and a negative correlation between soil pH and the relative abundance of *Xanthobacteraceae*. These results demonstrated that pH was an important factor in the transformation of microbial communities by RDA and strongly related to microbial richness and diversity (Kim et al., 2016). There are a number of soil characteristics (e.g., nutrient availability, cationic metal solubility, and organic C characteristics) that are often directly or indirectly related to soil pH, and these factors may drive the observed changes in community composition (Bissett et al., 2011; Suleiman et al., 2013). High soil salinity is an important factor leading to soil salinization that occurs easily, particularly due to the heavy use of fertilizers (Shi et al., 2009). Previous studies found that, as a consequence, soil salinity (indicated by EC) markedly decreased after RSD treatment, which was inconsistent with our results (Huang et al., 2016b). This dramatic increase was possibly driven by the decrease of nirK-, nirS-, and nosZ-type denitrifies in soil with the extension of planting time.

Furthermore, the effects of different treatments on trace elements in soil and their transformation are not consistent across different studies. In our study, SFC and RSD treatments increased the contents of AK, AZn, TNa, TFe, TCu, and TZn, while decreasing the contents of ACa, ASr, and TMn compared with CK. Likewise, RDA also showed that TCu was positively correlated with the relative abundances of *norank o_ Gaiellales* and *Sphingomonadaceae* and negatively correlated with the relative abundance of *Xanthobacteraceae*. The relative abundance of TNa was positively correlated with *Sphingomonadaceae*, *Gemmatimonadaceae*, *Bacillaceae*, and *Xanthobacteraceae*. This change may be caused directly by chloropicrin and anaerobic degradation of animal feces or indirectly by nutrient cycling (Zhao et al., 2018; Zhang et al., 2021). In short, change of soil properties depends on soil background and type of material used (Di Gioia et al., 2017).

Soil enzyme is important for organic substrate decomposition and biogeochemical cycling and is a useful biological indicator of soil functions (Fioretto et al., 2000; Xu et al., 2010). Soil enzyme activity is closely related to soil properties and bacterial community. In our study, the activities of S-ACP, S-CAT, and SL were significantly lower than those in the CK treatment except S-UE and S-SC, and they were in the order of CK > RSD > SFC. Further analysis showed that S-ACP and S-SC were positively correlated with the relative abundances of *norank o_ Gaiellales* and *Xanthobacteraceae*, and S-UE was positively correlated with the relative abundance of *Sphingomonadaceae*. However, the relative abundances of S-ACP and S-SC were negatively correlated with *Sphingomonadaceae*, *Gemmatimonadaceae*, and *Bacillaceae*, and S-UE was negatively correlated with the relative abundance of *Xanthobacteraceae*. This may depend on soil properties (especially pH and contents of available and total trace elements) and the specificity of different microorganisms to soil enzyme activities. Most enzymes in soil are secreted by soil microorganisms (Ahmed et al., 2018). However, bacteria that act as activators of some enzymes may act as inhibitors of others (Park et al., 2021). At the same time, when the abundance of the same flora is different, it can both activate and inhibit (Hu et al., 2021).

Therefore, it is still a complex and difficult task to fully clarify the driving factors and mechanisms of diversity and composition of the soil bacterial community. Further studies are needed to link the observed changes in the structure of soil microbial communities with soil functionality and to determine the core microbial community that would allow maintenance of at least some soil ecosystem services.

**FIGURE 8:**
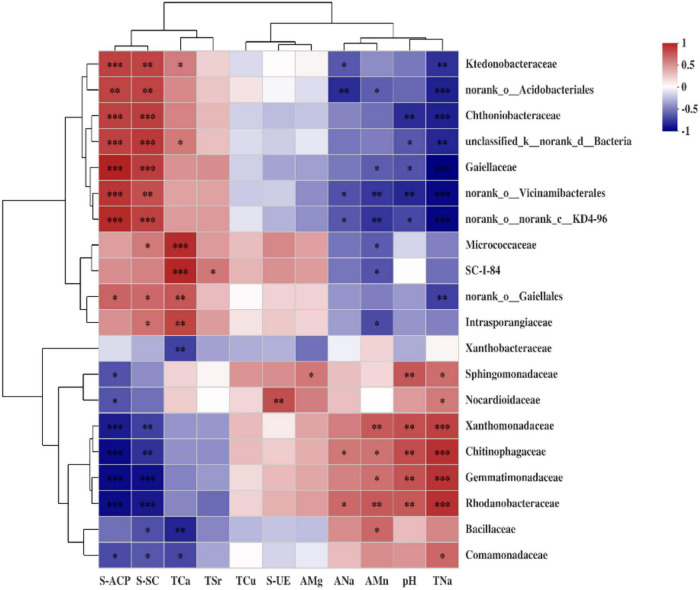
Heat map of the Spearman correlation coefficient between soil environmental factors and abundant bacterial family. Each value represents the mean of four replicates. The *, ^**^, and ^***^ represent 0.01 < *p* ≤ 0.05, 0.001 < *p* ≤ 0.01, and *p* ≤ 0.001, respectively.

Furthermore, the top 20 bacterial family clusters could be divided into 6 subclusters (Figure 8). Subclusters 1 and 2 were positively correlated with S-ACP, S-SC, and TCa, while being negatively correlated with ANa, AMn, pH, and TNa. However, Subclusters 4, 5, and 6 were negatively correlated with S-ACP, S-SC, and TCa, while being positively correlated with ANa, AMn, pH, and TNa. Subcluster 3 for *Xanthobacteraceae* has demonstrated a significant negative correlation with TCa.

**FIGURE 9:**
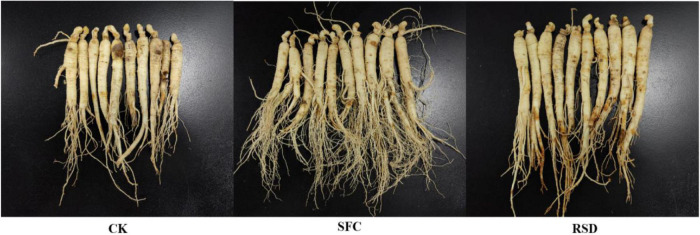
Root morphology of the ginseng under different treatments.

**FIGURE 10:**
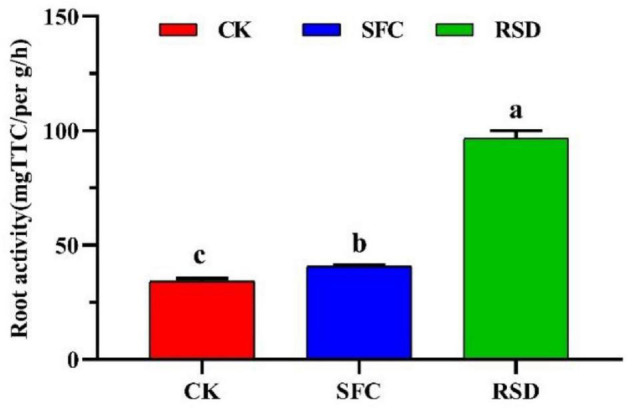
Root activity of the ginseng under different treatments. Error bars indicate the standard errors of the means of three replicates. The letters indicate significant difference at *p* < 0.05 according to one-way analysis of variance (ANOVA) among treatments.

### Reductive Soil Disinfestation Practice Incorporated With Organic Residue Combination Had Synergetic Effects on Soil Functionality Restoration and Plant Growth More Than Soil Fumigant Chloropicrin

Changes in bacterial community composition mainly depend on shifts in soil microbial diversity, and bacterial community composition-driven enhancements of soil nutrient cycles are typically associated with high levels of microbial diversity (Chen et al., 2010; Campos et al., 2014). In this study, SFC and RSD treatments dramatically altered core bacterial microbiomes and, similarly, the 20 most abundant bacterial families. Strikingly, we observed that the bacterial families *Comamonadaceae*, *Chitinophagaceae*, *Xanthobacteraceae*, *Rhodanobacteraceae*, *Bacillaceae*, *Gemmatimonadaceae*, *Sphingomonadaceae*, and *Nocardioidaceae* were considerably enriched in the SFC-treated soil. Members of the genus *Bacillus*, belonging to the family *Bacillaceae*, are known to produce various antibiotics, including lipopeptides and bacillibactin, that suppress a wide range of soil-borne plant pathogens (Xu et al., 2002; Li et al., 2014). Numerous studies have demonstrated that antibacterial substances produced by *Bacillus* can control a variety of plant diseases. Some biocontrol strains of *Bacillus* have been commercialized or licensed for limited commercial production and application (Wang et al., 2013). Interestingly, we observed that the bacterial families *Sphingomonadaceae*, *Micrococcaceae*, and *Nocardioidaceae* were considerably enriched in the SFC-treated soil. *Sphingomonadaceae* is known to be a class of microorganisms that can degrade PAHs and phenols and can survive under poor and harsh conditions with good environmental adaptability and tolerance (Baboshin et al., 2008; Liu et al., 2018).

In addition, the abundance, diversity, and activity of specific microbial taxa are also important factors in determining specific soil functions (Silva et al., 2004). Therefore, manipulation of the core microbiome for a given soil is a potential strategy for achieving the desired soil functionalities that could be beneficial for plant health and productivity (Chaparro et al., 2012). In this study, the majority of the shared OTUs was classified into 46 bacterial families, and the relative abundance of 42 families shifted considerably among treatments. It is likely due to the fact that the combination of different organic residues could stimulate a combined group of microbial taxa, thus resulting in higher microbial diversities. Meanwhile, *Methylophilaceae* and *Nocardioidaceae* were found only in the unique microbiomes of the SFC-treated soil, whereas *Alicyclobacillaceae* was found only in the unique microbiomes of the RSD-treated soil. Oxygen and nitrate are important determinants of microbial community structure. *Methylophilaceae* is the dominant microbial type in oxygen-rich culture environments containing nitrate (Beck et al., 2013; Chistoserdova et al., 2013). *Nocardioidaceae* and *Alicyclobacillaceae* members of these two families are reported to be linked to decomposition activity (Packard et al., 2019; Yoon et al., 2021). Previous studies have also found that the fungal genera *Penicillium* and *Chaetomium*, which are important decomposers, were enriched in the unique microbiomes of RSD-treated soil (Zhao et al., 2018; Huang et al., 2019). These results corresponded with our research.

Furthermore, studies have shown that plants change the composition of the soil community, and this change must then, in turn, affect the rate of growth of the plant or population (Bever, 1994). Microbial community is an indicator of soil health and quality (Schloter et al., 2003; Feng et al., 2019). A healthy soil will guarantee normal growth of plants. SFC and RSD are considered to be efficient pre-planting management practices to alleviate detrimental soil chemical properties and control soil-borne diseases. However, whether they can benefit the growth of crop plants is not yet known. Our results indicated that CK-treated ginseng root had more disease spots and lower root activity. However, after the application of SFC and RSD technology, the growth of ginseng was facilitated, and root performance was improved, which is consistent with previous studies (Yang et al., 2021), but there are significant differences between the effects of the two treatments. In general, the root morphology of ginseng treated with SFC was better, and the root activity of ginseng treated with RSD was stronger. Generally speaking, the pathogens that infect the root of ginseng mostly invade in the case of poor growth of ginseng or root injury. This may be because the untreated ginseng root lacks the protection of mechanical tissue and is prone to infection of pathogens and harm by adverse factors in the growth process, which is also the main reason for serious ginseng root disease (Li et al., 2021). However, some diseases are caused by some harmful substances in the soil. At the beginning, the damage of these diseases is serious, but later, the harmful substances are gradually degraded, and the diseases are also reduced or even disappeared, which is closely related to the mechanism of RSD technology. A large number of studies have shown that the interactions among plant roots, soil bacteria, and soil properties modulate plant performance by promoting or suppressing soil-borne pathogens, soil organic matter decomposition, and nutrient circulation and utilization (Wang et al., 2018; Feng et al., 2019). We speculate that the altered soil properties and enzyme activity inevitably affected soil microorganisms, soil animals, and plant roots. Meanwhile, a change in soil enzymes in turn affects soil microorganisms, the transformation of soil nutrients, and root growth.

While SFC has major effects on the soil microbial community, these effects decrease slowly over time, as reinfestation by pathogens occurs after host plant cultivation. Therefore, RSD can be used as a potential agricultural practice for the development of resistant soils. Although the effect of RSD on agricultural sustainability is intriguing, more attention needs to be given to the persistence of RSD effects on soil properties and host plant growth.

## Conclusion

We investigated the different responses of soil environmental factors, soil bacterial community, and root performance to RSD and SFC. This research provides evidence that both SFC and RSD treatments could improve soil properties and root performance *via* the alteration of bacterial microbiomes. RSD-treated soils, incorporated with organic residues, harbored distinct bacterial microbiomes with lower bacterial richness, diversity, and evenness. In addition, they exhibited higher functional richness and diversity compared with SFC with chloropicrin-treated soil. Moreover, RSD alleviated unfavorable soil properties, fostering more diverse disease-suppressive and organic-decomposable agents, restructured the bacterial community, and improved root performance. Thus, RSD is an effective and environmentally friendly pre-planting management practice to counteract some of the negative effects of ginseng production on soil. It can be applied as a potential agricultural practice for the development of disease-suppressive soil.

## Data Availability Statement

The datasets presented in this study can be found in online repositories. The names of the repository/repositories and accession number(s) can be found below: https://www.ncbi.nlm.nih.gov/, PRJNA765568.

## Author Contributions

All authors listed have made a substantial, direct, and intellectual contribution to the work, and approved it for publication.

## Conflict of Interest

The authors declare that the research was conducted in the absence of any commercial or financial relationships that could be construed as a potential conflict of interest.

## Publisher’s Note

All claims expressed in this article are solely those of the authors and do not necessarily represent those of their affiliated organizations, or those of the publisher, the editors and the reviewers. Any product that may be evaluated in this article, or claim that may be made by its manufacturer, is not guaranteed or endorsed by the publisher.
